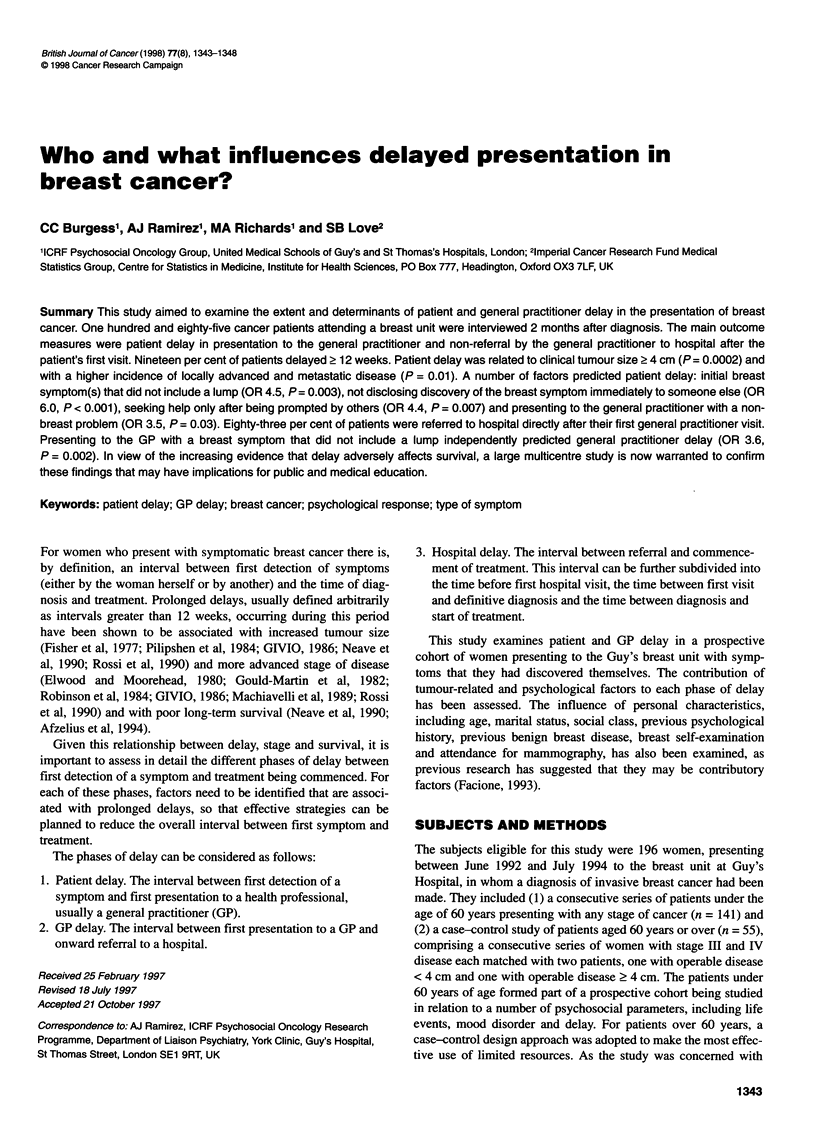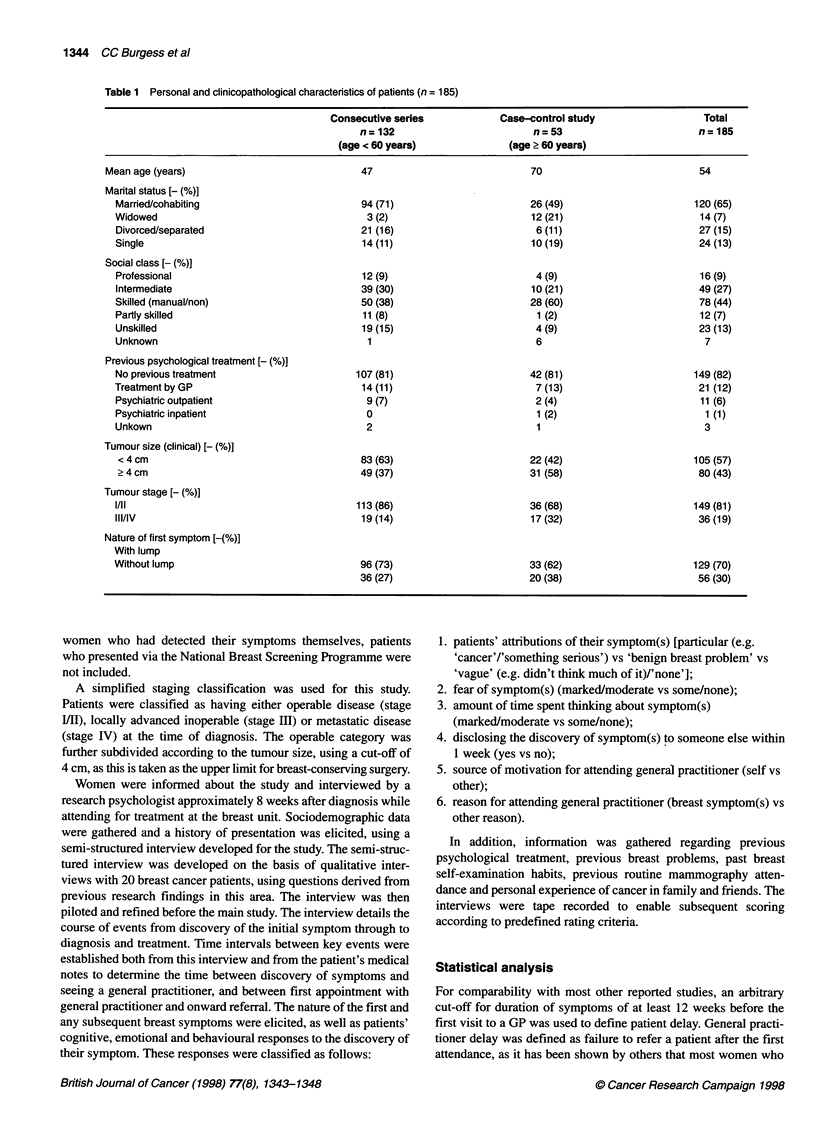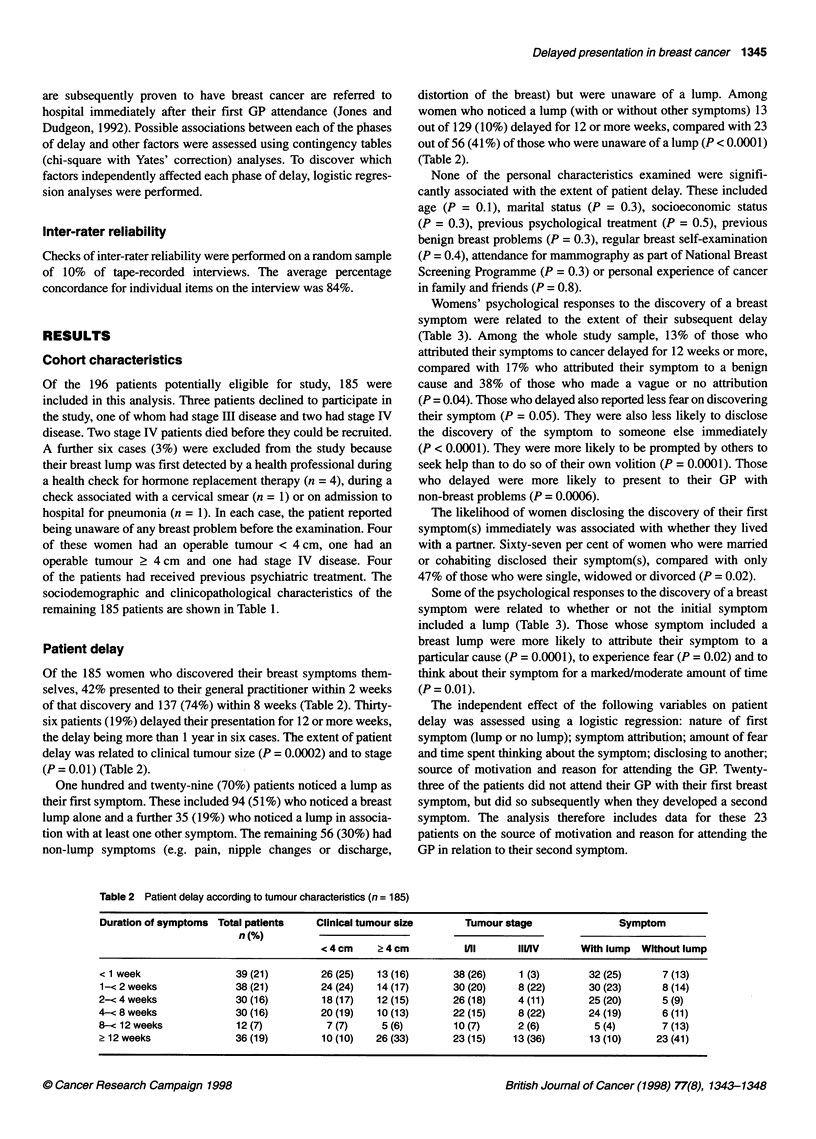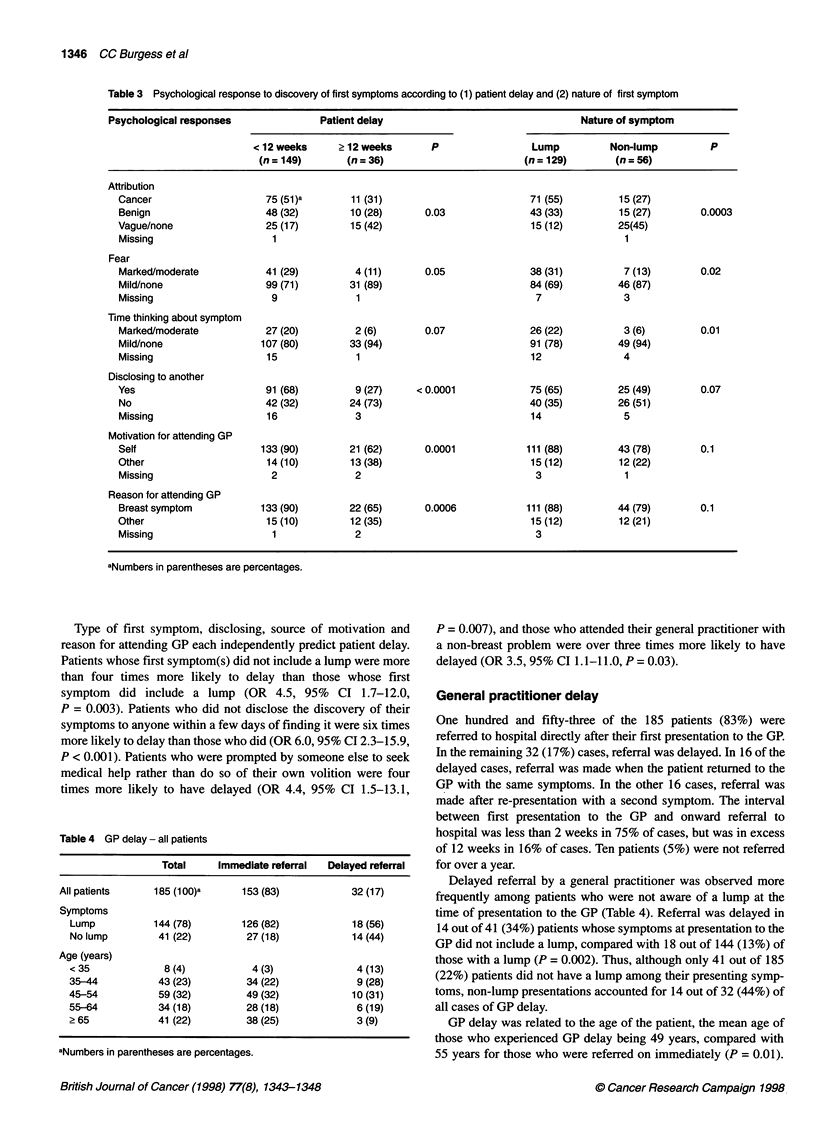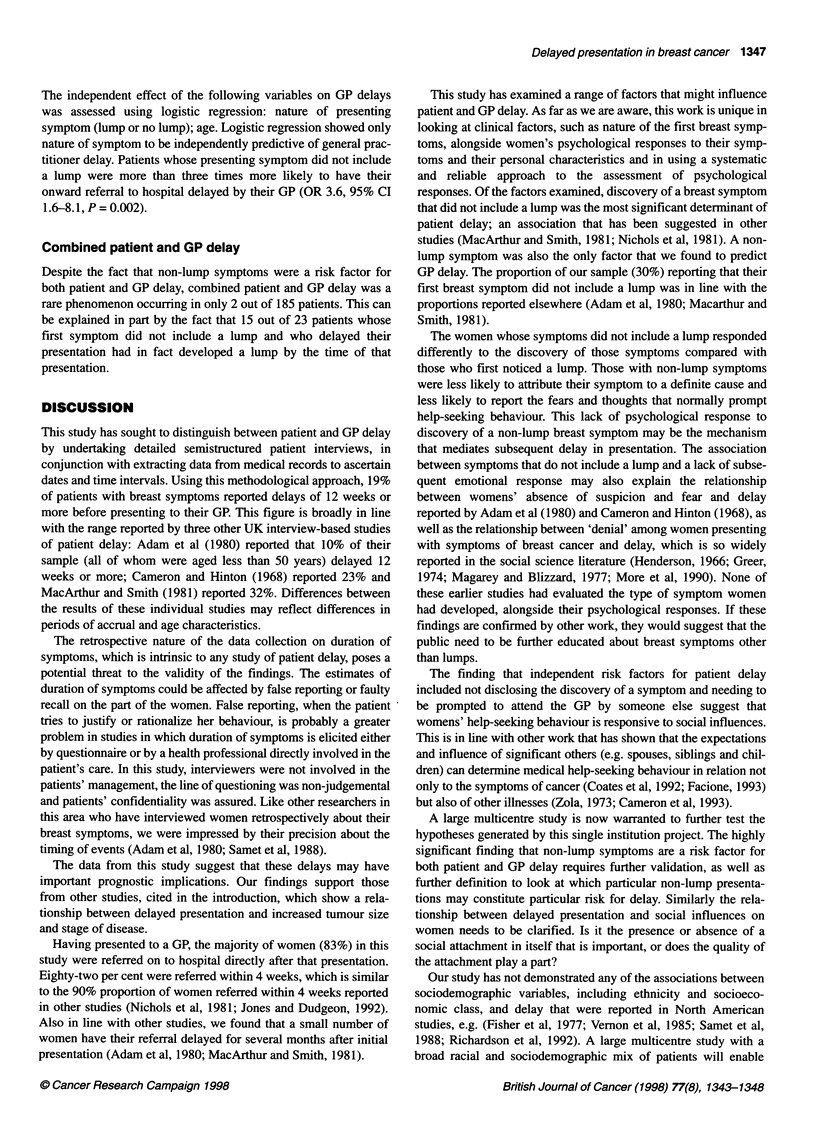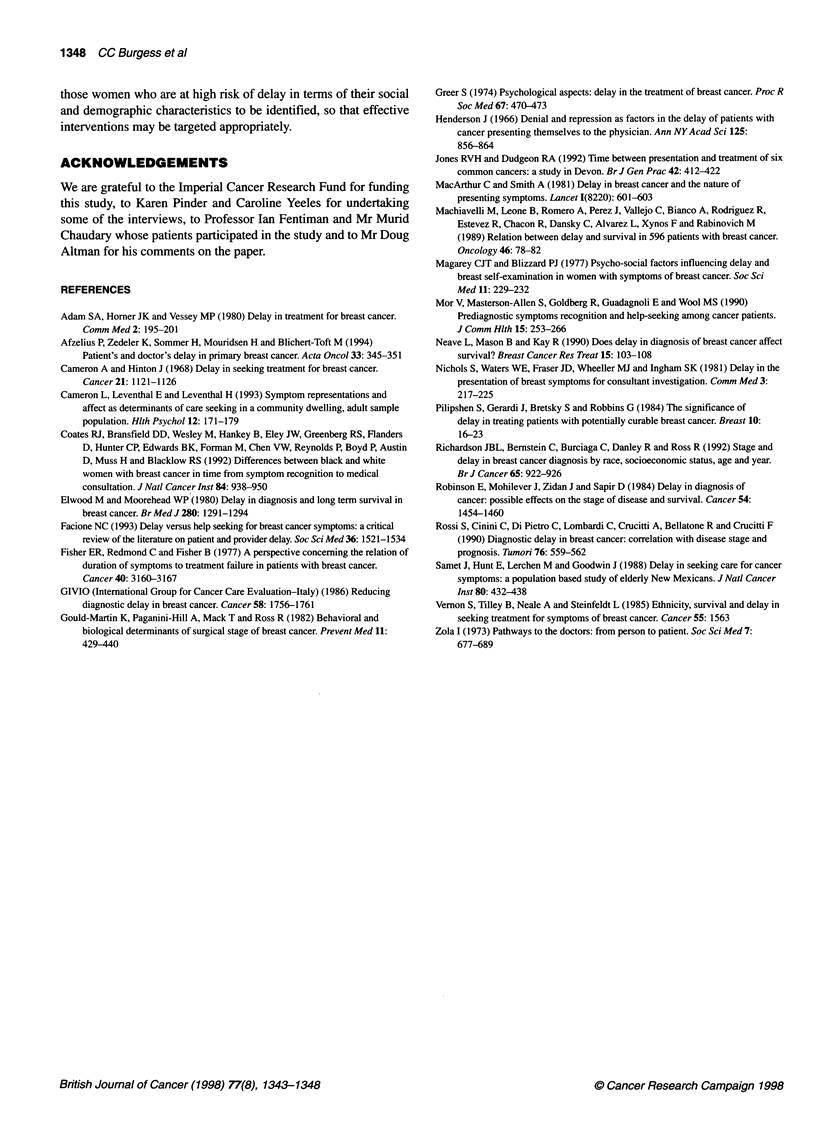# Who and what influences delayed presentation in breast cancer?

**DOI:** 10.1038/bjc.1998.224

**Published:** 1998-04

**Authors:** C. C. Burgess, A. J. Ramirez, M. A. Richards, S. B. Love

**Affiliations:** ICRF Psychosocial Oncology Group, United Medical School of Guy's Hospital, London, UK.

## Abstract

This study aimed to examine the extent and determinants of patient and general practitioner delay in the presentation of breast cancer. One hundred and eighty-five cancer patients attending a breast unit were interviewed 2 months after diagnosis. The main outcome measures were patient delay in presentation to the general practitioner and non-referral by the general practitioner to hospital after the patient's first visit. Nineteen per cent of patients delayed > or = 12 weeks. Patient delay was related to clinical tumour size > or = 4 cm (P = 0.0002) and with a higher incidence of locally advanced and metastatic disease (P = 0.01). A number of factors predicted patient delay: initial breast symptom(s) that did not include a lump (OR 4.5, P = 0.003), not disclosing discovery of the breast symptom immediately to someone else (OR 6.0, P < 0.001), seeking help only after being prompted by others (OR 4.4, P = 0.007) and presenting to the general practitioner with a non-breast problem (OR 3.5, P = 0.03). Eighty-three per cent of patients were referred to hospital directly after their first general practitioner visit. Presenting to the GP with a breast symptom that did not include a lump independently predicted general practitioner delay (OR 3.6, P = 0.002). In view of the increasing evidence that delay adversely affects survival, a large multicentre study is now warranted to confirm these findings that may have implications for public and medical education.


British Journal of Cancer (1998) 77(8), 1343–1348
© 1998 Cancer Research Campaign

### Who and what influences delayed presentation in breast cancer?

**CC Burgess[1], AJ Ramirez[1], MA Richards[1] and SB Love[2]**

[1]ICRF Psychosocial Oncology Group, United Medical Schools of Guy's and St Thomas's Hospitals, London; [2]Imperial Cancer Research Fund Medical Statistics Group, Centre for Statistics in Medicine, Institute for Health Sciences, PO Box 777, Headington, Oxford OX3 7LF, UK

**Summary** This study aimed to examine the extent and determinants of patient and general practitioner delay in the presentation of breast cancer. One hundred and eighty-five cancer patients attending a breast unit were interviewed 2 months after diagnosis. The main outcome measures were patient delay in presentation to the general practitioner and non-referral by the general practitioner to hospital after the patient's first visit. Nineteen per cent of patients delayed ≥ 12 weeks. Patient delay was related to clinical tumour size ≥ 4 cm ($P = 0.0002$) and with a higher incidence of locally advanced and metastatic disease ($P = 0.01$). A number of factors predicted patient delay: initial breast symptom(s) that did not include a lump (OR 4.5, $P = 0.003$), not disclosing discovery of the breast symptom immediately to someone else (OR 6.0, $P < 0.001$), seeking help only after being prompted by others (OR 4.4, $P = 0.007$) and presenting to the general practitioner with a non-breast problem (OR 3.5, $P = 0.03$). Eighty-three per cent of patients were referred to hospital directly after their first general practitioner visit. Presenting to the GP with a breast symptom that did not include a lump independently predicted general practitioner delay (OR 3.6, $P = 0.002$). In view of the increasing evidence that delay adversely affects survival, a large multicentre study is now warranted to confirm these findings that may have implications for public and medical education.

**Keywords:** patient delay; GP delay; breast cancer; psychological response; type of symptom

For women who present with symptomatic breast cancer there is, by definition, an interval between first detection of symptoms (either by the woman herself or by another) and the time of diagnosis and treatment. Prolonged delays, usually defined arbitrarily as intervals greater than 12 weeks, occurring during this period have been shown to be associated with increased tumour size (Fisher et al, 1977; Pilipshen et al, 1984; GIVIO, 1986; Neave et al, 1990; Rossi et al, 1990) and more advanced stage of disease (Elwood and Moorehead, 1980; Gould-Martin et al, 1982; Robinson et al, 1984; GIVIO, 1986; Machiavelli et al, 1989; Rossi et al, 1990) and with poor long-term survival (Neave et al, 1990; Afzelius et al, 1994).

Given this relationship between delay, stage and survival, it is important to assess in detail the different phases of delay between first detection of a symptom and treatment being commenced. For each of these phases, factors need to be identified that are associated with prolonged delays, so that effective strategies can be planned to reduce the overall interval between first symptom and treatment.

The phases of delay can be considered as follows:

1. Patient delay. The interval between first detection of a symptom and first presentation to a health professional, usually a general practitioner (GP).
2. GP delay. The interval between first presentation to a GP and onward referral to a hospital.
3. Hospital delay. The interval between referral and commencement of treatment. This interval can be further subdivided into the time before first hospital visit, the time between first visit and definitive diagnosis and the time between diagnosis and start of treatment.

This study examines patient and GP delay in a prospective cohort of women presenting to the Guy's breast unit with symptoms that they had discovered themselves. The contribution of tumour-related and psychological factors to each phase of delay has been assessed. The influence of personal characteristics, including age, marital status, social class, previous psychological history, previous benign breast disease, breast self-examination and attendance for mammography, has also been examined, as previous research has suggested that they may be contributory factors (Facione, 1993).

### SUBJECTS AND METHODS

The subjects eligible for this study were 196 women, presenting between June 1992 and July 1994 to the breast unit at Guy's Hospital, in whom a diagnosis of invasive breast cancer had been made. They included (1) a consecutive series of patients under the age of 60 years presenting with any stage of cancer ($n = 141$) and (2) a case–control study of patients aged 60 years or over ($n = 55$), comprising a consecutive series of women with stage III and IV disease each matched with two patients, one with operable disease < 4 cm and one with operable disease ≥ 4 cm. The patients under 60 years of age formed part of a prospective cohort being studied in relation to a number of psychosocial parameters, including life events, mood disorder and delay. For patients over 60 years, a case–control design approach was adopted to make the most effective use of limited resources. As the study was concerned with

*Received 25 February 1997*
*Revised 18 July 1997*
*Accepted 21 October 1997*

*Correspondence to:* AJ Ramirez, ICRF Psychosocial Oncology Research Programme, Department of Liaison Psychiatry, York Clinic, Guy's Hospital, St Thomas Street, London SE1 9RT, UK

**Table 1** Personal and clinicopathological characteristics of patients ($n$ = 185)

| | Consecutive series $n$ = 132 (age < 60 years) | Case–control study $n$ = 53 (age ≥ 60 years) | Total $n$ = 185 |
|---|---|---|---|
| Mean age (years) | 47 | 70 | 54 |
| Marital status [– (%)] | | | |
| Married/cohabiting | 94 (71) | 26 (49) | 120 (65) |
| Widowed | 3 (2) | 12 (21) | 14 (7) |
| Divorced/separated | 21 (16) | 6 (11) | 27 (15) |
| Single | 14 (11) | 10 (19) | 24 (13) |
| Social class [– (%)] | | | |
| Professional | 12 (9) | 4 (9) | 16 (9) |
| Intermediate | 39 (30) | 10 (21) | 49 (27) |
| Skilled (manual/non) | 50 (38) | 28 (60) | 78 (44) |
| Partly skilled | 11 (8) | 1 (2) | 12 (7) |
| Unskilled | 19 (15) | 4 (9) | 23 (13) |
| Unknown | 1 | 6 | 7 |
| Previous psychological treatment [– (%)] | | | |
| No previous treatment | 107 (81) | 42 (81) | 149 (82) |
| Treatment by GP | 14 (11) | 7 (13) | 21 (12) |
| Psychiatric outpatient | 9 (7) | 2 (4) | 11 (6) |
| Psychiatric inpatient | 0 | 1 (2) | 1 (1) |
| Unkown | 2 | 1 | 3 |
| Tumour size (clinical) [– (%)] | | | |
| < 4 cm | 83 (63) | 22 (42) | 105 (57) |
| ≥ 4 cm | 49 (37) | 31 (58) | 80 (43) |
| Tumour stage [– (%)] | | | |
| I/II | 113 (86) | 36 (68) | 149 (81) |
| III/IV | 19 (14) | 17 (32) | 36 (19) |
| Nature of first symptom [–(%)] | | | |
| With lump | | | |
| Without lump | 96 (73) | 33 (62) | 129 (70) |
| | 36 (27) | 20 (38) | 56 (30) |

women who had detected their symptoms themselves, patients who presented via the National Breast Screening Programme were not included.

A simplified staging classification was used for this study. Patients were classified as having either operable disease (stage I/II), locally advanced inoperable (stage III) or metastatic disease (stage IV) at the time of diagnosis. The operable category was further subdivided according to the tumour size, using a cut-off of 4 cm, as this is taken as the upper limit for breast-conserving surgery.

Women were informed about the study and interviewed by a research psychologist approximately 8 weeks after diagnosis while attending for treatment at the breast unit. Sociodemographic data were gathered and a history of presentation was elicited, using a semi-structured interview developed for the study. The semi-structured interview was developed on the basis of qualitative interviews with 20 breast cancer patients, using questions derived from previous research findings in this area. The interview was then piloted and refined before the main study. The interview details the course of events from discovery of the initial symptom through to diagnosis and treatment. Time intervals between key events were established both from this interview and from the patient's medical notes to determine the time between discovery of symptoms and seeing a general practitioner, and between first appointment with general practitioner and onward referral. The nature of the first and any subsequent breast symptoms were elicited, as well as patients' cognitive, emotional and behavioural responses to the discovery of their symptom. These responses were classified as follows:

1. patients' attributions of their symptom(s) [particular (e.g. 'cancer'/'something serious') vs 'benign breast problem' vs 'vague' (e.g. didn't think much of it)/'none'];
2. fear of symptom(s) (marked/moderate vs some/none);
3. amount of time spent thinking about symptom(s) (marked/moderate vs some/none);
4. disclosing the discovery of symptom(s) to someone else within 1 week (yes vs no);
5. source of motivation for attending general practitioner (self vs other);
6. reason for attending general practitioner (breast symptom(s) vs other reason).

In addition, information was gathered regarding previous psychological treatment, previous breast problems, past breast self-examination habits, previous routine mammography attendance and personal experience of cancer in family and friends. The interviews were tape recorded to enable subsequent scoring according to predefined rating criteria.

### Statistical analysis

For comparability with most other reported studies, an arbitrary cut-off for duration of symptoms of at least 12 weeks before the first visit to a GP was used to define patient delay. General practitioner delay was defined as failure to refer a patient after the first attendance, as it has been shown by others that most women who

 © Cancer Research Campaign 1998

are subsequently proven to have breast cancer are referred to hospital immediately after their first GP attendance (Jones and Dudgeon, 1992). Possible associations between each of the phases of delay and other factors were assessed using contingency tables (chi-square with Yates' correction) analyses. To discover which factors independently affected each phase of delay, logistic regression analyses were performed.

### Inter-rater reliability

Checks of inter-rater reliability were performed on a random sample of 10% of tape-recorded interviews. The average percentage concordance for individual items on the interview was 84%.

### RESULTS

### Cohort characteristics

Of the 196 patients potentially eligible for study, 185 were included in this analysis. Three patients declined to participate in the study, one of whom had stage III disease and two had stage IV disease. Two stage IV patients died before they could be recruited. A further six cases (3%) were excluded from the study because their breast lump was first detected by a health professional during a health check for hormone replacement therapy ($n$ = 4), during a check associated with a cervical smear ($n$ = 1) or on admission to hospital for pneumonia ($n$ = 1). In each case, the patient reported being unaware of any breast problem before the examination. Four of these women had an operable tumour < 4 cm, one had an operable tumour ≥ 4 cm and one had stage IV disease. Four of the patients had received previous psychiatric treatment. The sociodemographic and clinicopathological characteristics of the remaining 185 patients are shown in Table 1.

### Patient delay

Of the 185 women who discovered their breast symptoms themselves, 42% presented to their general practitioner within 2 weeks of that discovery and 137 (74%) within 8 weeks (Table 2). Thirty-six patients (19%) delayed their presentation for 12 or more weeks, the delay being more than 1 year in six cases. The extent of patient delay was related to clinical tumour size ($P$ = 0.0002) and to stage ($P$ = 0.01) (Table 2).

One hundred and twenty-nine (70%) patients noticed a lump as their first symptom. These included 94 (51%) who noticed a breast lump alone and a further 35 (19%) who noticed a lump in association with at least one other symptom. The remaining 56 (30%) had non-lump symptoms (e.g. pain, nipple changes or discharge, distortion of the breast) but were unaware of a lump. Among women who noticed a lump (with or without other symptoms) 13 out of 129 (10%) delayed for 12 or more weeks, compared with 23 out of 56 (41%) of those who were unaware of a lump ($P$ < 0.0001) (Table 2).

None of the personal characteristics examined were significantly associated with the extent of patient delay. These included age ($P$ = 0.1), marital status ($P$ = 0.3), socioeconomic status ($P$ = 0.3), previous psychological treatment ($P$ = 0.5), previous benign breast problems ($P$ = 0.3), regular breast self-examination ($P$ = 0.4), attendance for mammography as part of National Breast Screening Programme ($P$ = 0.3) or personal experience of cancer in family and friends ($P$ = 0.8).

Womens' psychological responses to the discovery of a breast symptom were related to the extent of their subsequent delay (Table 3). Among the whole study sample, 13% of those who attributed their symptoms to cancer delayed for 12 weeks or more, compared with 17% who attributed their symptom to a benign cause and 38% of those who made a vague or no attribution ($P$ = 0.04). Those who delayed also reported less fear on discovering their symptom ($P$ = 0.05). They were also less likely to disclose the discovery of the symptom to someone else immediately ($P$ < 0.0001). They were more likely to be prompted by others to seek help than to do so of their own volition ($P$ = 0.0001). Those who delayed were more likely to present to their GP with non-breast problems ($P$ = 0.0006).

The likelihood of women disclosing the discovery of their first symptom(s) immediately was associated with whether they lived with a partner. Sixty-seven per cent of women who were married or cohabiting disclosed their symptom(s), compared with only 47% of those who were single, widowed or divorced ($P$ = 0.02).

Some of the psychological responses to the discovery of a breast symptom were related to whether or not the initial symptom included a lump (Table 3). Those whose symptom included a breast lump were more likely to attribute their symptom to a particular cause ($P$ = 0.0001), to experience fear ($P$ = 0.02) and to think about their symptom for a marked/moderate amount of time ($P$ = 0.01).

The independent effect of the following variables on patient delay was assessed using a logistic regression: nature of first symptom (lump or no lump); symptom attribution; amount of fear and time spent thinking about the symptom; disclosing to another; source of motivation and reason for attending the GP. Twenty-three of the patients did not attend their GP with their first breast symptom, but did so subsequently when they developed a second symptom. The analysis therefore includes data for these 23 patients on the source of motivation and reason for attending the GP in relation to their second symptom.

**Table 2** Patient delay according to tumour characteristics ($n$ = 185)

| Duration of symptoms | Total patients $n$ (%) | Clinical tumour size | | Tumour stage | | Symptom | |
|---|---|---|---|---|---|---|---|
| | | < 4 cm | ≥ 4 cm | I/II | III/IV | With lump | Without lump |
| < 1 week | 39 (21) | 26 (25) | 13 (16) | 38 (26) | 1 (3) | 32 (25) | 7 (13) |
| 1–< 2 weeks | 38 (21) | 24 (24) | 14 (17) | 30 (20) | 8 (22) | 30 (23) | 8 (14) |
| 2–< 4 weeks | 30 (16) | 18 (17) | 12 (15) | 26 (18) | 4 (11) | 25 (20) | 5 (9) |
| 4–< 8 weeks | 30 (16) | 20 (19) | 10 (13) | 22 (15) | 8 (22) | 24 (19) | 6 (11) |
| 8–< 12 weeks | 12 (7) | 7 (7) | 5 (6) | 10 (7) | 2 (6) | 5 (4) | 7 (13) |
| ≥ 12 weeks | 36 (19) | 10 (10) | 26 (33) | 23 (15) | 13 (36) | 13 (10) | 23 (41) |

© Cancer Research Campaign 1998

**Table 3** Psychological response to discovery of first symptoms according to (1) patient delay and (2) nature of first symptom

| Psychological responses | Patient delay | | | Nature of symptom | | |
|---|---|---|---|---|---|---|
| | < 12 weeks (*n* = 149) | ≥ 12 weeks (*n* = 36) | *P* | Lump (*n* = 129) | Non-lump (*n* = 56) | *P* |
| Attribution | | | | | | |
| Cancer | 75 (51)[a] | 11 (31) | | 71 (55) | 15 (27) | |
| Benign | 48 (32) | 10 (28) | 0.03 | 43 (33) | 15 (27) | 0.0003 |
| Vague/none | 25 (17) | 15 (42) | | 15 (12) | 25(45) | |
| Missing | 1 | | | | 1 | |
| Fear | | | | | | |
| Marked/moderate | 41 (29) | 4 (11) | 0.05 | 38 (31) | 7 (13) | 0.02 |
| Mild/none | 99 (71) | 31 (89) | | 84 (69) | 46 (87) | |
| Missing | 9 | 1 | | 7 | 3 | |
| Time thinking about symptom | | | | | | |
| Marked/moderate | 27 (20) | 2 (6) | 0.07 | 26 (22) | 3 (6) | 0.01 |
| Mild/none | 107 (80) | 33 (94) | | 91 (78) | 49 (94) | |
| Missing | 15 | 1 | | 12 | 4 | |
| Disclosing to another | | | | | | |
| Yes | 91 (68) | 9 (27) | < 0.0001 | 75 (65) | 25 (49) | 0.07 |
| No | 42 (32) | 24 (73) | | 40 (35) | 26 (51) | |
| Missing | 16 | 3 | | 14 | 5 | |
| Motivation for attending GP | | | | | | |
| Self | 133 (90) | 21 (62) | 0.0001 | 111 (88) | 43 (78) | 0.1 |
| Other | 14 (10) | 13 (38) | | 15 (12) | 12 (22) | |
| Missing | 2 | 2 | | 3 | 1 | |
| Reason for attending GP | | | | | | |
| Breast symptom | 133 (90) | 22 (65) | 0.0006 | 111 (88) | 44 (79) | 0.1 |
| Other | 15 (10) | 12 (35) | | 15 (12) | 12 (21) | |
| Missing | 1 | 2 | | 3 | | |

[a]Numbers in parentheses are percentages.

Type of first symptom, disclosing, source of motivation and reason for attending GP each independently predict patient delay. Patients whose first symptom(s) did not include a lump were more than four times more likely to delay than those whose first symptom did include a lump (OR 4.5, 95% CI 1.7–12.0, $P$ = 0.003). Patients who did not disclose the discovery of their symptoms to anyone within a few days of finding it were six times more likely to delay than those who did (OR 6.0, 95% CI 2.3–15.9, $P$ < 0.001). Patients who were prompted by someone else to seek medical help rather than do so of their own volition were four times more likely to have delayed (OR 4.4, 95% CI 1.5–13.1, $P$ = 0.007), and those who attended their general practitioner with a non-breast problem were over three times more likely to have delayed (OR 3.5, 95% CI 1.1–11.0, $P$ = 0.03).

**Table 4** GP delay – all patients

| | Total | Immediate referral | Delayed referral |
|---|---|---|---|
| All patients | 185 (100)[a] | 153 (83) | 32 (17) |
| Symptoms | | | |
| Lump | 144 (78) | 126 (82) | 18 (56) |
| No lump | 41 (22) | 27 (18) | 14 (44) |
| Age (years) | | | |
| < 35 | 8 (4) | 4 (3) | 4 (13) |
| 35–44 | 43 (23) | 34 (22) | 9 (28) |
| 45–54 | 59 (32) | 49 (32) | 10 (31) |
| 55–64 | 34 (18) | 28 (18) | 6 (19) |
| ≥ 65 | 41 (22) | 38 (25) | 3 (9) |

[a]Numbers in parentheses are percentages.

### General practitioner delay

One hundred and fifty-three of the 185 patients (83%) were referred to hospital directly after their first presentation to the GP. In the remaining 32 (17%) cases, referral was delayed. In 16 of the delayed cases, referral was made when the patient returned to the GP with the same symptoms. In the other 16 cases, referral was made after re-presentation with a second symptom. The interval between first presentation to the GP and onward referral to hospital was less than 2 weeks in 75% of cases, but was in excess of 12 weeks in 16% of cases. Ten patients (5%) were not referred for over a year.

Delayed referral by a general practitioner was observed more frequently among patients who were not aware of a lump at the time of presentation to the GP (Table 4). Referral was delayed in 14 out of 41 (34%) patients whose symptoms at presentation to the GP did not include a lump, compared with 18 out of 144 (13%) of those with a lump ($P$ = 0.002). Thus, although only 41 out of 185 (22%) patients did not have a lump among their presenting symptoms, non-lump presentations accounted for 14 out of 32 (44%) of all cases of GP delay.

GP delay was related to the age of the patient, the mean age of those who experienced GP delay being 49 years, compared with 55 years for those who were referred on immediately ($P$ = 0.01).

© Cancer Research Campaign 1998

The independent effect of the following variables on GP delays was assessed using logistic regression: nature of presenting symptom (lump or no lump); age. Logistic regression showed only nature of symptom to be independently predictive of general practitioner delay. Patients whose presenting symptom did not include a lump were more than three times more likely to have their onward referral to hospital delayed by their GP (OR 3.6, 95% CI 1.6–8.1, $P = 0.002$).

### Combined patient and GP delay

Despite the fact that non-lump symptoms were a risk factor for both patient and GP delay, combined patient and GP delay was a rare phenomenon occurring in only 2 out of 185 patients. This can be explained in part by the fact that 15 out of 23 patients whose first symptom did not include a lump and who delayed their presentation had in fact developed a lump by the time of that presentation.

### DISCUSSION

This study has sought to distinguish between patient and GP delay by undertaking detailed semistructured patient interviews, in conjunction with extracting data from medical records to ascertain dates and time intervals. Using this methodological approach, 19% of patients with breast symptoms reported delays of 12 weeks or more before presenting to their GP. This figure is broadly in line with the range reported by three other UK interview-based studies of patient delay: Adam et al (1980) reported that 10% of their sample (all of whom were aged less than 50 years) delayed 12 weeks or more; Cameron and Hinton (1968) reported 23% and MacArthur and Smith (1981) reported 32%. Differences between the results of these individual studies may reflect differences in periods of accrual and age characteristics.

The retrospective nature of the data collection on duration of symptoms, which is intrinsic to any study of patient delay, poses a potential threat to the validity of the findings. The estimates of duration of symptoms could be affected by false reporting or faulty recall on the part of the women. False reporting, when the patient tries to justify or rationalize her behaviour, is probably a greater problem in studies in which duration of symptoms is elicited either by questionnaire or by a health professional directly involved in the patient's care. In this study, interviewers were not involved in the patients' management, the line of questioning was non-judgemental and patients' confidentiality was assured. Like other researchers in this area who have interviewed women retrospectively about their breast symptoms, we were impressed by their precision about the timing of events (Adam et al, 1980; Samet et al, 1988).

The data from this study suggest that these delays may have important prognostic implications. Our findings support those from other studies, cited in the introduction, which show a relationship between delayed presentation and increased tumour size and stage of disease.

Having presented to a GP, the majority of women (83%) in this study were referred on to hospital directly after that presentation. Eighty-two per cent were referred within 4 weeks, which is similar to the 90% proportion of women referred within 4 weeks reported in other studies (Nichols et al, 1981; Jones and Dudgeon, 1992). Also in line with other studies, we found that a small number of women have their referral delayed for several months after initial presentation (Adam et al, 1980; MacArthur and Smith, 1981).

This study has examined a range of factors that might influence patient and GP delay. As far as we are aware, this work is unique in looking at clinical factors, such as nature of the first breast symptoms, alongside women's psychological responses to their symptoms and their personal characteristics and in using a systematic and reliable approach to the assessment of psychological responses. Of the factors examined, discovery of a breast symptom that did not include a lump was the most significant determinant of patient delay; an association that has been suggested in other studies (MacArthur and Smith, 1981; Nichols et al, 1981). A non-lump symptom was also the only factor that we found to predict GP delay. The proportion of our sample (30%) reporting that their first breast symptom did not include a lump was in line with the proportions reported elsewhere (Adam et al, 1980; Macarthur and Smith, 1981).

The women whose symptoms did not include a lump responded differently to the discovery of those symptoms compared with those who first noticed a lump. Those with non-lump symptoms were less likely to attribute their symptom to a definite cause and less likely to report the fears and thoughts that normally prompt help-seeking behaviour. This lack of psychological response to discovery of a non-lump breast symptom may be the mechanism that mediates subsequent delay in presentation. The association between symptoms that do not include a lump and a lack of subsequent emotional response may also explain the relationship between womens' absence of suspicion and fear and delay reported by Adam et al (1980) and Cameron and Hinton (1968), as well as the relationship between 'denial' among women presenting with symptoms of breast cancer and delay, which is so widely reported in the social science literature (Henderson, 1966; Greer, 1974; Magarey and Blizzard, 1977; More et al, 1990). None of these earlier studies had evaluated the type of symptom women had developed, alongside their psychological responses. If these findings are confirmed by other work, they would suggest that the public need to be further educated about breast symptoms other than lumps.

The finding that independent risk factors for patient delay included not disclosing the discovery of a symptom and needing to be prompted to attend the GP by someone else suggest that womens' help-seeking behaviour is responsive to social influences. This is in line with other work that has shown that the expectations and influence of significant others (e.g. spouses, siblings and children) can determine medical help-seeking behaviour in relation not only to the symptoms of cancer (Coates et al, 1992; Facione, 1993) but also of other illnesses (Zola, 1973; Cameron et al, 1993).

A large multicentre study is now warranted to further test the hypotheses generated by this single institution project. The highly significant finding that non-lump symptoms are a risk factor for both patient and GP delay requires further validation, as well as further definition to look at which particular non-lump presentations may constitute particular risk for delay. Similarly the relationship between delayed presentation and social influences on women needs to be clarified. Is it the presence or absence of a social attachment in itself that is important, or does the quality of the attachment play a part?

Our study has not demonstrated any of the associations between sociodemographic variables, including ethnicity and socioeconomic class, and delay that were reported in North American studies, e.g. (Fisher et al, 1977; Vernon et al, 1985; Samet et al, 1988; Richardson et al, 1992). A large multicentre study with a broad racial and sociodemographic mix of patients will enable

© Cancer Research Campaign 1998 

those women who are at high risk of delay in terms of their social and demographic characteristics to be identified, so that effective interventions may be targeted appropriately.

### ACKNOWLEDGEMENTS

We are grateful to the Imperial Cancer Research Fund for funding this study, to Karen Pinder and Caroline Yeeles for undertaking some of the interviews, to Professor Ian Fentiman and Mr Murid Chaudary whose patients participated in the study and to Mr Doug Altman for his comments on the paper.

### REFERENCES

Adam SA, Horner JK and Vessey MP (1980) Delay in treatment for breast cancer. *Comm Med* **2**: 195–201

Afzelius P, Zedeler K, Sommer H, Mouridsen H and Blichert-Toft M (1994) Patient's and doctor's delay in primary breast cancer. *Acta Oncol* **33**: 345–351

Cameron A and Hinton J (1968) Delay in seeking treatment for breast cancer. *Cancer* **21**: 1121–1126

Cameron L, Leventhal E and Leventhal H (1993) Symptom representations and affect as determinants of care seeking in a community dwelling, adult sample population. *Hlth Psychol* **12**: 171–179

Coates RJ, Bransfield DD, Wesley M, Hankey B, Eley JW, Greenberg RS, Flanders D, Hunter CP, Edwards BK, Forman M, Chen VW, Reynolds P, Boyd P, Austin D, Muss H and Blacklow RS (1992) Differences between black and white women with breast cancer in time from symptom recognition to medical consultation. *J Natl Cancer Inst* **84**: 938–950

Elwood M and Moorehead WP (1980) Delay in diagnosis and long term survival in breast cancer. *Br Med J* **280**: 1291–1294

Facione NC (1993) Delay versus help seeking for breast cancer symptoms: a critical review of the literature on patient and provider delay. *Soc Sci Med* **36**: 1521–1534

Fisher ER, Redmond C and Fisher B (1977) A perspective concerning the relation of duration of symptoms to treatment failure in patients with breast cancer. *Cancer* **40**: 3160–3167

GIVIO (International Group for Cancer Care Evaluation–Italy) (1986) Reducing diagnostic delay in breast cancer. *Cancer* **58**: 1756–1761

Gould-Martin K, Paganini-Hill A, Mack T and Ross R (1982) Behavioral and biological determinants of surgical stage of breast cancer. *Prevent Med* **11**: 429–440

Greer S (1974) Psychological aspects: delay in the treatment of breast cancer. *Proc R Soc Med* **67**: 470–473

Henderson J (1966) Denial and repression as factors in the delay of patients with cancer presenting themselves to the physician. *Ann NY Acad Sci* **125**: 856–864

Jones RVH and Dudgeon RA (1992) Time between presentation and treatment of six common cancers: a study in Devon. *Br J Gen Prac* **42**: 412–422

MacArthur C and Smith A (1981) Delay in breast cancer and the nature of presenting symptoms. *Lancet* **I**(8220): 601–603

Machiavelli M, Leone B, Romero A, Perez J, Vallejo C, Bianco A, Rodriguez R, Estevez R, Chacon R, Dansky C, Alvarez L, Xynos F and Rabinovich M (1989) Relation between delay and survival in 596 patients with breast cancer. *Oncology* **46**: 78–82

Magarey CJT and Blizzard PJ (1977) Psycho-social factors influencing delay and breast self-examination in women with symptoms of breast cancer. *Soc Sci Med* **11**: 229–232

Mor V, Masterson-Allen S, Goldberg R, Guadagnoli E and Wool MS (1990) Prediagnostic symptoms recognition and help-seeking among cancer patients. *J Comm Hlth* **15**: 253–266

Neave L, Mason B and Kay R (1990) Does delay in diagnosis of breast cancer affect survival? *Breast Cancer Res Treat* **15**: 103–108

Nichols S, Waters WE, Fraser JD, Wheeller MJ and Ingham SK (1981) Delay in the presentation of breast symptoms for consultant investigation. *Comm Med* **3**: 217–225

Pilipshen S, Gerardi J, Bretsky S and Robbins G (1984) The significance of delay in treating patients with potentially curable breast cancer. *Breast* **10**: 16–23

Richardson JBL, Bernstein C, Burciaga C, Danley R and Ross R (1992) Stage and delay in breast cancer diagnosis by race, socioeconomic status, age and year. *Br J Cancer* **65**: 922–926

Robinson E, Mohilever J, Zidan J and Sapir D (1984) Delay in diagnosis of cancer: possible effects on the stage of disease and survival. *Cancer* **54**: 1454–1460

Rossi S, Cinini C, Di Pietro C, Lombardi C, Crucitti A, Bellatone R and Crucitti F (1990) Diagnostic delay in breast cancer: correlation with disease stage and prognosis. *Tumori* **76**: 559–562

Samet J, Hunt E, Lerchen M and Goodwin J (1988) Delay in seeking care for cancer symptoms: a population based study of elderly New Mexicans. *J Natl Cancer Inst* **80**: 432–438

Vernon S, Tilley B, Neale A and Steinfeldt L (1985) Ethnicity, survival and delay in seeking treatment for symptoms of breast cancer. *Cancer* **55**: 1563

Zola I (1973) Pathways to the doctors: from person to patient. *Soc Sci Med* **7**: 677–689

© Cancer Research Campaign 1998